# Microstructural and plasmonic modifications in Ag–TiO_2_ and Au–TiO_2_ nanocomposites through ion beam irradiation

**DOI:** 10.3762/bjnano.5.154

**Published:** 2014-09-01

**Authors:** Venkata Sai Kiran Chakravadhanula, Yogendra Kumar Mishra, Venkata Girish Kotnur, Devesh Kumar Avasthi, Thomas Strunskus, Vladimir Zaporotchenko, Dietmar Fink, Lorenz Kienle, Franz Faupel

**Affiliations:** 1 Chair for Multicomponent Materials, Institute for Materials Science, Christian Albrechts University Kiel, Kaiserstr. 2, Kiel, 24143, Germany; 2 Functional Nanomaterials, Institute for Materials Science, Christian Albrechts University Kiel, Kaiserstr. 2, Kiel, 24143, Germany; 3 Inter University Accelerator Center, Materials Science Group, P.O. Box: 10502, New Delhi, 110067, India; 4 Instituto da Fisica, Universidad Autonoma Metropolitana–Iztapalapa, Av. San Rafael Atlixco No. 186, Col. Vicentina, Delegacion Iztapalapa, Mexico D.F., 09340, Mexico; 5 Synthesis and Real Structure, Institute for Materials Science, Christian Albrechts University Kiel, Kaiserstr. 2, Kiel, 24143, Germany

**Keywords:** noble metal–titania nanocomposite, surface plasmon resonance, swift heavy ions

## Abstract

The development of new fabrication techniques of plasmonic nanocomposites with specific properties is an ongoing issue in the plasmonic and nanophotonics community. In this paper we report detailed investigations on the modifications of the microstructural and plasmonic properties of metal–titania nanocomposite films induced by swift heavy ions. Au–TiO_2_ and Ag–TiO_2_ nanocomposite thin films with varying metal volume fractions were deposited by co-sputtering and were subsequently irradiated by 100 MeV Ag^8+^ ions at various ion fluences. The morphology of these nanocomposite thin films before and after ion beam irradiation has been investigated in detail by transmission electron microscopy studies, which showed interesting changes in the titania matrix. Additionally, interesting modifications in the plasmonic absorption behavior for both Au–TiO_2_ and Ag–TiO_2_ nanocomposites were observed, which have been discussed in terms of ion beam induced growth of nanoparticles and structural modifications in the titania matrix.

## Introduction

Metal nanoparticles embedded in dielectric matrices in the form of nanocomposites have gained significant research interest due their multifunctional properties appropriate for various applications ranging from solar cells to targeted drug delivery [1–4]. The plasmonic properties of the nanocomposite films mainly depend upon the type of nanoparticles (Au or Ag), their morphology and the dielectric constant of the embedding matrix [5–6]. As the dielectric constant in the expression for extinction coefficient (denominator), hence the refractive index of the matrix plays a very important role in surface plasmon resonance (SPR). Several dielectric matrices, such as SiO_2_ and polymers have been utilized to fabricate different multifunctional nanocomposites for different applications [7–9]. Generally, the main motivation behind the use of an insulating matrix is to maintain the necessary separation between metal nanoparticles (resulting from differences in surface energy of the individual components), thereby preventing an agglomeration of the metallic nanoparticles. However, further functionalities are added to the nanocomposite system if semiconducting matrices are used, in which the dielectric properties of the matrix allows for a better tunability of SPR. In this regard, the use of semiconducting matrices, such as SnO_2_ [10], ZnO [11] and CdS [12] for the embedding of noble metal nanoparticles has shown great potential.

Thin films and nanostructures of TiO_2_ are probably one of the most investigated systems for different applications, such as memristors, dye-sensitized solar cells, antibacterial coatings, photocatalysts, and implants [13–18]. The different properties of metal–TiO_2_ nanocomposites mainly depend on the metal volume filling fraction and the stoichiometry of the matrix. Generally, once the nanocomposites are prepared their properties are fixed. It is therefore very difficult to further modify the plasmonic response of these already synthesized nanocomposites. An additional fabrication experiment with slightly modified parameters might help. In this regards, the use of swift heavy ions (SHI) in order to modify the properties of the prepared nanocomposites in a controlled manner by selecting appropriate ion energies and fluences is a promising alternative [19]. The use of SHI has already shown its potential for controlling the morphology of the metal nanoparticles embedded in a silica matrix [20–26]. So far, in these experiments the chosen matrix was silica because of the fact that the effect of swift heavy ion irradiation of silica in terms of creating an ion track is well understood [27–29]. To summarize, the nanoparticles grow in size if they are close to each other and their sizes are smaller than the diameter of ion track, whereas if the inter particle distance is larger a size reduction occurs. If the particles are larger than the diameter of ion track, but smaller than a particular size, they elongate along the ion beam direction, resulting in parallel elongated nanoparticles [22,27,30–32]. SHI irradiation can result in reduction, growth, or elongation of nanoparticles in a controlled manner and thereby facilitating the tuning of the SPR wavelength of the nanocomposite system. In the scenario described here, the aim was to study the swift heavy ion irradiation of noble metal nanoparticles embedded in a matrix, in which the formation of ion tracks is not known to occur. Under this premise, we picked a TiO_2_ matrix. Unlike silica, SHI irradiation might introduce several other types of structural changes in the TiO_2_ matrix, which in turn affect the plasmonic properties of the nanocomposite system [17]. The detailed structural modifications and changes of optical properties of pure titania thin films under SHI irradiations have been already investigated [17,33–37]. Detailed understandings about the modification of metal–SiO_2_ and metal–polymer nanocomposites under SHI irradiation have already been reported but such studies about metal–TiO_2_ nanocomposites would be very interesting. Titania is a wide band gap semiconductor, and the tuning of the SPR in such a matrix by ion beam irradiation is another aim of the present work. Hence, the effects of swift heavy ion irradiation on metal–TiO_2_ nanocomposites at different ion beam fluences has been studied and discussed here.

## Results and Discussion

**Figure 1:**
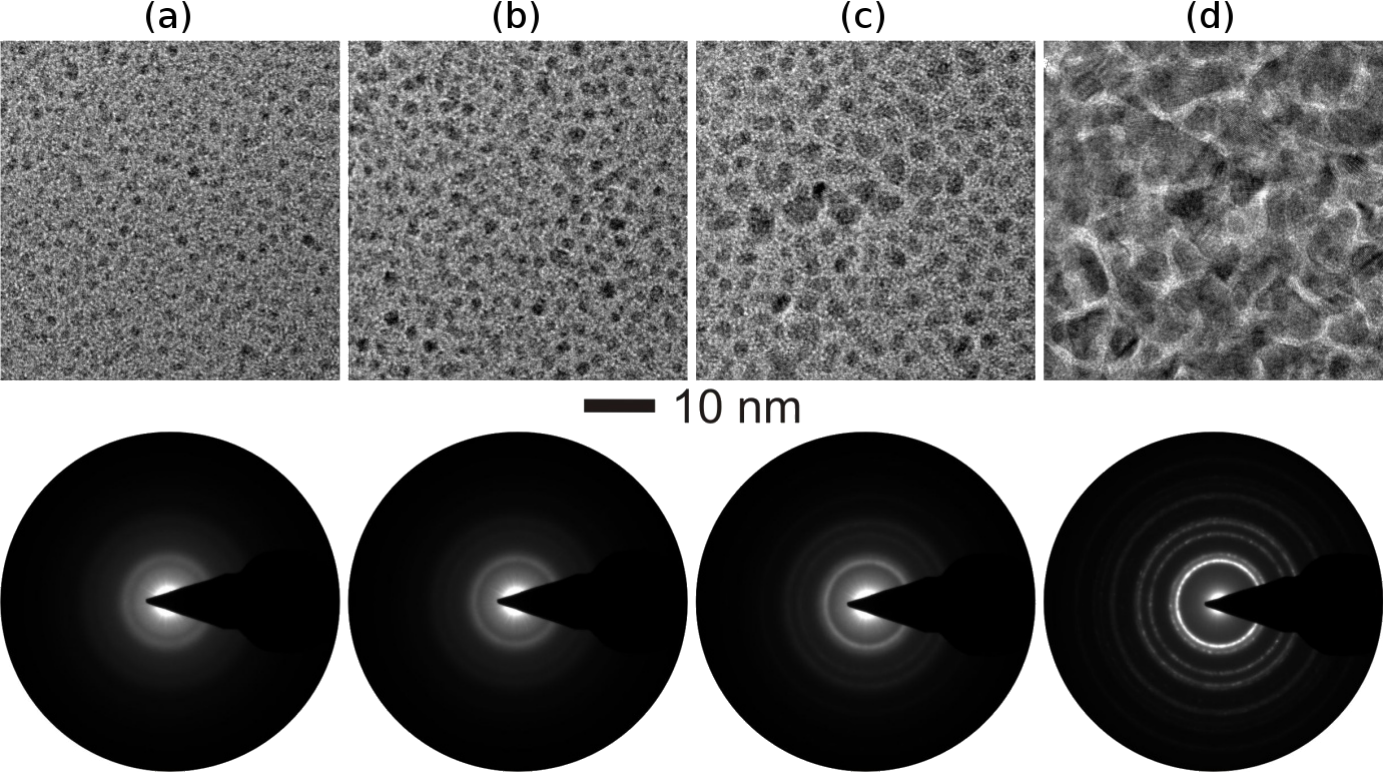
Bright-field TEM images of Au–TiO_2_ nanocomposite thin films with different MVFs, (a) 7%, (b) 11%, (c) 13% and (d) 50%. In these bright-field TEM images, dark and bright areas correspond to the Au nanoparticles and the TiO_2_ matrix, respectively. The selected area electron diffraction (SAED) patterns corresponding to each MVF composite is shown exactly below each TEM image.

The microstructural morphologies of Au–TiO_2_ nanocomposites with metal volume filling fractions (MVF) from 7 to 50% were investigated by transmission electron microscopy (TEM) studies and are shown in Figure 1. With the increase of the Au MVF from 7 to 13%, the average diameter of the Au nanoparticles increased and for an extreme case, in which the Au MVF was about 50%, the growth of extremely large nanoparticles has been observed (Figure 1d). The selected area electron diffraction patterns corresponding to each nanocomposite film are shown below the bright-field TEM images. They demonstrate that the TiO_2_ matrix in the nanocomposite film is in an amorphous state.

**Figure 2:**
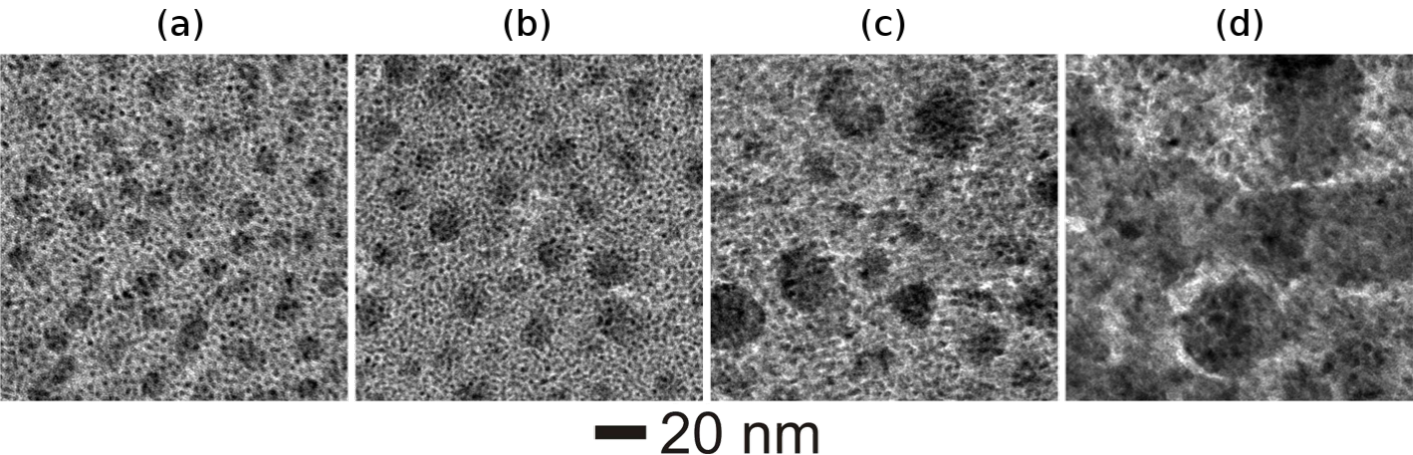
Bright field TEM morphologies of Ag–TiO_2_ nanocomposite films with different metal volume filling fractions, (a) 15%, (b) 26%, (c) 34% and (d) 47%.

In similar manner, Ag–TiO_2_ nanocomposite thin films with varying Ag MVF (from 15 to 47%) have been synthesized and the corresponding bright-field TEM images are shown in Figure 2. A closer look at all TEM images in Figure 2 reveals the growth of smaller as well as larger Ag nanoparticles during co-sputtering process and the average diameter of Ag nanoparticles increases with increasing Ag metal volume fraction. In fact a deeper look at the TEM images of Au–TiO_2_ nanocomposites (Figure 1) also confirmed the growth of smaller Au nanoparticles apart from the clearly visible ones (those with dark contrast in the bright field TEM images). Such type of Ag nanoparticle growth has also been observed in other matrices, e.g., SiO_2_ [38]. The dark and bright contrasts in the TEM image correspond to Ag nanoparticles and TiO_2_ matrix, respectively.

Detailed investigations on the particle size distribution of the Ag nanoparticles embedded in a TiO_2_ matrix have been performed by 3D-tomography studies [39–40]. Tomography results have confirmed the bimodal distribution of Ag nanoparticles with the presence of larger nanoparticles on top of the surface and smaller nanoparticles embedded inside the matrix.

To investigate the effect of ion irradiation on metal–TiO_2_ nanocomposites, the deposited Au–TiO_2_ and Ag–TiO_2_ nanocomposite films (both with MVF ≈ 15%) were selected. Nanocomposite films with 15% metal volume fraction were intentionally chosen because of the intermediate values of inter-particle separation (IPS) between the metal nanoparticles. Nanoparticle size and inter-particle separation are the two very important parameters responsible for dissolution, growth or elongation of nanoparticles due to SHI irradiation. For a nanocomposite with relatively small nanoparticle diameter (smaller than the ion track diameter) and larger IPS, the dissolution of nanoparticles occurs due to SHI irradiation [22]. However if the IPS distance is very low, a growth of nanoparticles occurs under ion irradiation irrespective of the particle diameter. Elongation of metal nanoparticles along the ion beam direction in the nanocomposite has been observed mostly for the cases when the average diameter of nanoparticles was equal to or larger than the ion track diameters [25]. The host matrix of the nanocomposite film plays a very important role during swift heavy ion irradiation. Due to unpredictive nature of the TiO_2_ matrix, Au–TiO_2_ (MVF ≈ 15%) and Ag–TiO_2_ (MVF ≈ 15%) nanocomposites were selected for study as in both nanocomposites isolated nanoparticles embedded in TiO_2_ matrix can be observed (Figure 1 and Figure 2) and the IPS distances are also not too large.

**Figure 3:**
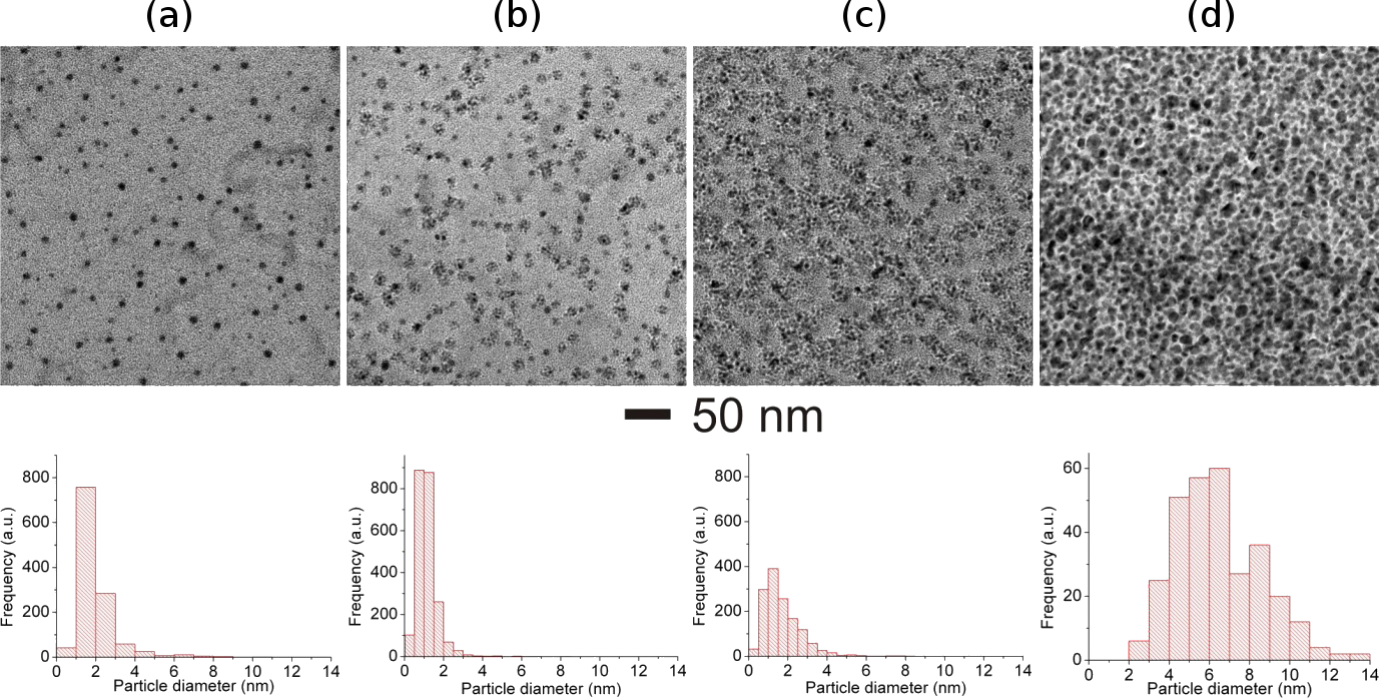
Morphological evolutions in Au–TiO_2_ nanocomposite (MVF ≈ 15%) under 100 MeV Ag^8+^ ion irradiation at different fluences; Bright field TEM image of: (a) pristine film, (b) 1 × 10^12^ ions/cm^2^, (c) 3 × 10^12^ ions/cm^2^, (d) 1 × 10^13^ ions/cm^2^. Size distributions corresponding to each TEM image are shown below the images.

Bright-field TEM images of 100 MeV Ag^8+^ ion irradiated Au–TiO_2_ (MVF ≈ 15%) at different fluences are shown in Figure 3. In a pristine nanocomposite film, Au nanoparticles are well separated (Figure 3a) with average diameter of around 2 nm (see the size distribution corresponding to Figure 3a). An increase of the average diameter of Au nanoparticles from 2 to 7 nm has been observed after irradiation with fluences up to 1 × 10^13^ ions/cm^2^ as can be seen in bright-field TEM images (Figure 3b-d) and the corresponding size distributions.

The TEM image of the Au–TiO_2_ nanocomposite irradiated at the lowest fluence (1 × 10^12^ ions/cm^2^, Figure 3b) demonstrates the local growth growth of Au nanoparticles. The average diameter of the nanoparticle did not increase much but the density of nanoparticles has significantly increased. The local growth of the nanoparticle under SHI irradiation is attributed to the fact that the co-sputtered nanocomposite film exhibits a bi-modal distribution of nanoparticles. In the pristine sample along with visible nanoparticles (Figure 3a), single atoms, clusters and small nanoparticles of Au, which could not contribute to the nucleation and growth process, are also present, which could not contribute to nucleation and growth process. The electronic energy deposited by ions is converted into thermal energy, which enhances the process of nucleation and growth of metal nanoparticles in the nanocomposite film and hence more Au nanoparticles can be observed in the bright-field TEM image (in Figure 3b) corresponding to fluence 1 × 10^12^ ions/cm^2^. With an increase in ion fluence to 3 × 10^12^ ions/cm^2^, further growth of Au nanoparticles takes place (Figure 3c). The average diameter of the nanoparticles has not much increased but the particle size distribution has broadened. The diameter of some nanoparticles even exceeds 6 nm, with more nanoparticles (Figure 3c) in the size range from 2 to 6 nm as compared to pristine state (Figure 3a) and those irradiated at 1 × 10^12^ ions/cm^2^ (Figure 3b). It seems that an ion irradiation at about 3 × 10^12^ ions/cm^2^initiates the agglomeration of smaller nanoparticles. Thereby, the resultant number of Au nanoparticles having larger diameters has increased as compared to pristine and that irradiated at lower fluences. Since this fluence (about 3 × 10^12^ ions/cm^2^) almost corresponds to the track overlap value that results in the thermalization of the whole nanocomposite film, this kind of agglomeration (growth) behavior of Au nanoparticles in the Au–TiO_2_ film can be expected. The bright-field TEM image corresponding to the Au–TiO_2_ nanocomposite (Figure 3d) irradiated at a yet higher fluence (about 1 × 10^13^ ions/cm^2^) confirms the growth of large Au nanoparticles with diameters ranging up to 14 nm (size distribution in Figure 3d). Because of the agglomeration of the nanoparticles due to irradiation at high fluence, the particle density has been significantly reduced. Of course there exists a possibility of the sputtering of some metal nanoparticles from the surface of the nanocomposite due to ion irradiation. But it is very small and can be qualitatively ignored. However, accurate quantitative information requires precise ion beam experiments. It is important to emphasize here that there exists some nanoparticles with larger diameter in the pristine nanocomposite film (Figure 3c) which satisfy the condition of elongation [22]. However no elongation of nanoparticles been has been observed (conventional bright-field TEM image in Figure 3d) after irradiation up to a fluence of about 1 × 10^13^ ions/cm^2^. Despite the fact that condition for elongation (particle size ≥ track size) holds true, no elongation of nanoparticles under ion irradiation has been observed and it is probably due to absence of a latent track formation mechanism because of the semiconducting nature of the matrix as compared to insulating matrices (e.g., SiO_2_) in which ion tracks are usually formed [20,28–29,41].

**Figure 4:**
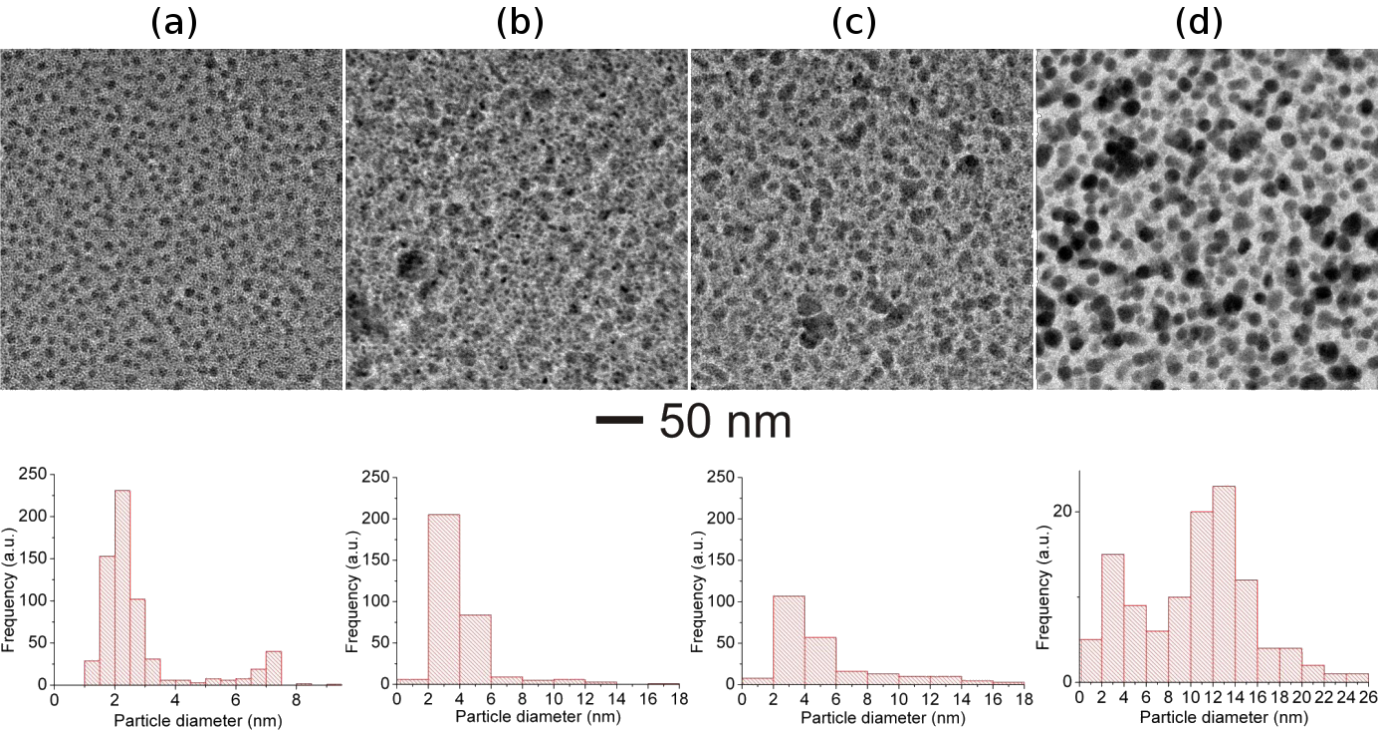
Morphological evolutions in Ag–TiO_2_ nanocomposite films (MVF ≈ 15%) under 100 MeV Ag^8+^ ion irradiation at different fluences. Bright-field TEM image of: (a) pristine film, (b) 1 × 10^12^ ions/cm^2^, (c) 3 × 10^12^ ions/cm^2^, (d) 1 × 10^13^ ions/cm^2^. The particle size distributions corresponding to each TEM image are shown below the images.

Ion irradiation studies on Ag–TiO_2_ nanocomposite (MVF ≈ 15%) film were also performed and corresponding bright-field TEM images are shown in Figure 4. The pristine Ag–TiO_2_ nanocomposite sample exhibits Ag nanoparticles with a bi-modal particle size distribution (Figure 4a and the corresponding particle size distribution) [39–40]. After irradiation with 100 MeV Ag^8+^ ions at a fluence of about 1 × 10^12^ ions/cm^2^, the average diameter of the Ag nanoparticles is increased indicating the growth of nanoparticles. A possible nanoparticle growth mechanism already discussed for Au–TiO_2_ nanocomposites in the previous section holds true. In contrast to Au–TiO_2_ system, the growth of Ag nanoparticles with relatively large diameters (Figure 4band corresponding particle size distribution) has been observed in Ag-TiO_2_ nanocomposites after SHI irradiation. After irradiating at a fluence of 3 × 10^12^ ions/cm^2^, a further growth of nanoparticles is observed and the density of the nanoparticles is reduced. This is obvious because smaller nanoparticles are agglomerated into bigger nanoparticles. Irradiation at the highest fluence of ca. 1 × 10^13^ ions/cm^2^ results in the growth of Ag nanoparticles with very large diameters (up to ca. 26 nm) with a broad size distribution (Figure 4dand its particle size distribution). Since the particle size is very large, the effective density of nanoparticles has been significantly decreased because formation of larger nanoparticles occurs only at the expense of smaller nanoparticles. Similar to the Au–TiO_2_ nanocomposites, no elongation of Ag nanoparticles in TiO_2_ matrix, apart from the large diameters, has been observed even at the highest fluence apart.

**Figure 5:**
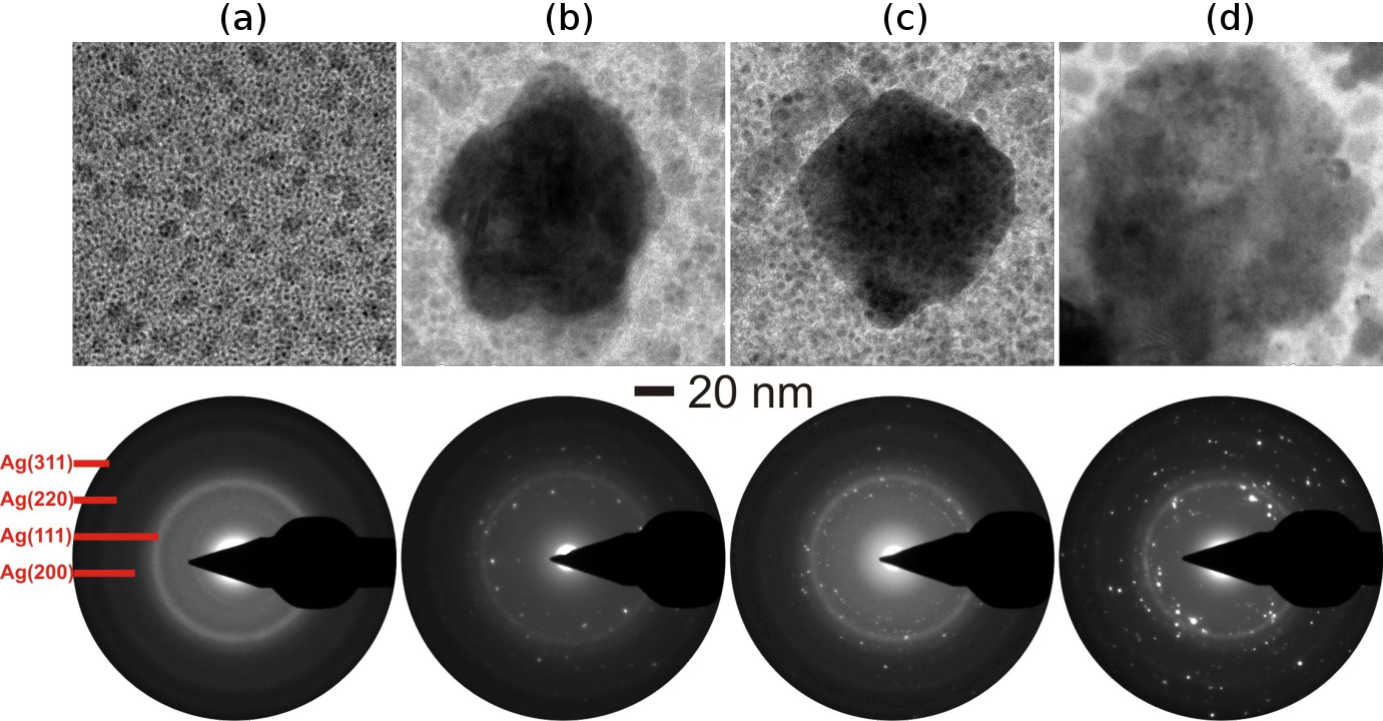
Microstructural changes in the TiO_2_ matrix of the nanocomposite film with MVF (Ag) ≈ 15% induced by 100 MeV Ag^8+^ ions. Bright field TEM images corresponding to: (a) pristine film, (b) 1 × 10^12^ ions/cm^2^, (c) 3 × 10^12^ ions/cm^2^, (d) 1 × 10^13^ ions/cm^2^. The selected area electron diffraction patterns with respect to pristine film (a) and different irradiation fluences are shown below. Crystallinity of the TiO_2_ matrix has been observed as a result of the reflections corresponding to the metrics from brookite and rutile structures from the SAED patterns from (b) to (d).

The growth of Au and Ag nanoparticles in a TiO_2_ matrix after ion irradiation with 100 MeV Ag^8+^ at different fluences has been demonstrated. However, the behavior of the TiO_2_ matrix under ion irradiation is extremely important. In fact the matrix of the nanocomposite film plays a very important role in reduction, growth and elongation of metal nanoparticles by swift heavy ion irradiation. When a swift heavy ion passes through the film, it deposits a large amount of electronic energy, which is instantly converted into thermal energy and thus each ion creates an ion track along its path. The large amount of thermal energy deposited by the ions results in a cylindrical zone along the ion path with very high temperatures. The corresponding temperature profile can be divided in two zones (i) the central zone, i.e., the ion path where the material is molten, and (ii) the surrounding zone where the matrix is not molten but the temperature is still high enough for metal nanoparticles to be in molten state. The formation of ion tracks in insulator matrices, e.g., SiO_2_, has been understood in terms of thermal spike and Coulomb explosion models [26,41–42]. But SHI-induced modifications in metal–semiconducting matrices like TiO_2_ are still unclear as changes in the matrix strongly affect the response of the metal nanoparticles to the ion irradiation. It is most probable that due to the semiconducting nature of TiO_2_, the formation of molten tracks does not occur and, hence, the elongation of nanoparticles is unexpected under SHI irradiation. However the large amount of electronic energy (*S*_e_) deposited by the ions in the nanocomposite film is sufficient for the growth of nanoparticles (Figure 3 and Figure 4) as well as other structural changes in the TiO_2_ matrix. In order to understand the SHI-induced effects, detailed microstructural studies of the Ag–TiO_2_ nanocomposite (MVF ≈ 15%) film irradiated at different fluences (1 × 10^12^ to 1 × 10^13^ ions/cm^2^) by using TEM and SAED analysis are shown in Figure 5.

The TiO_2_ matrix in the as-deposited (pristine) film is amorphous as revealed by SAED pattern corresponding to bright-field TEM image of Figure 5a. After irradiation at fluences of 1 × 10^12^, 3 × 10^12^ and 1 × 10^13^ ions/cm^2^, an increase of the crystallinity of the TiO_2_ matrix (metrics from brookite and rutile structures) has been observed from selected area electron diffraction patterns of Figure 5b–d. In addition, reflections corresponding to the metrics from TiO [43–44] were observed along with large TiO crystals after ion beam irradiation (see below in Figure 8 and Figure 9). Several studies on SHI-induced crystallization of amorphous TiO_2_ thin films have been performed and it has been reported that under SHI irradiation, the crystallization evolves through the formation of TiO_2_ nanocrystals in rutile and anatase phases [37,45]. In a similar study an increase of the dielectric constant of the TiO_2_ film after 100 MeV Ag^8+^ ion irradiation has been reported. This is another evidence for the increasing crystallinity [35,46]. SHI-induced crystallization in nanocomposite films plays indeed a very strong role in the growth behavior of embedded metal nanoparticles in the nanocomposite film.

**Figure 6:**
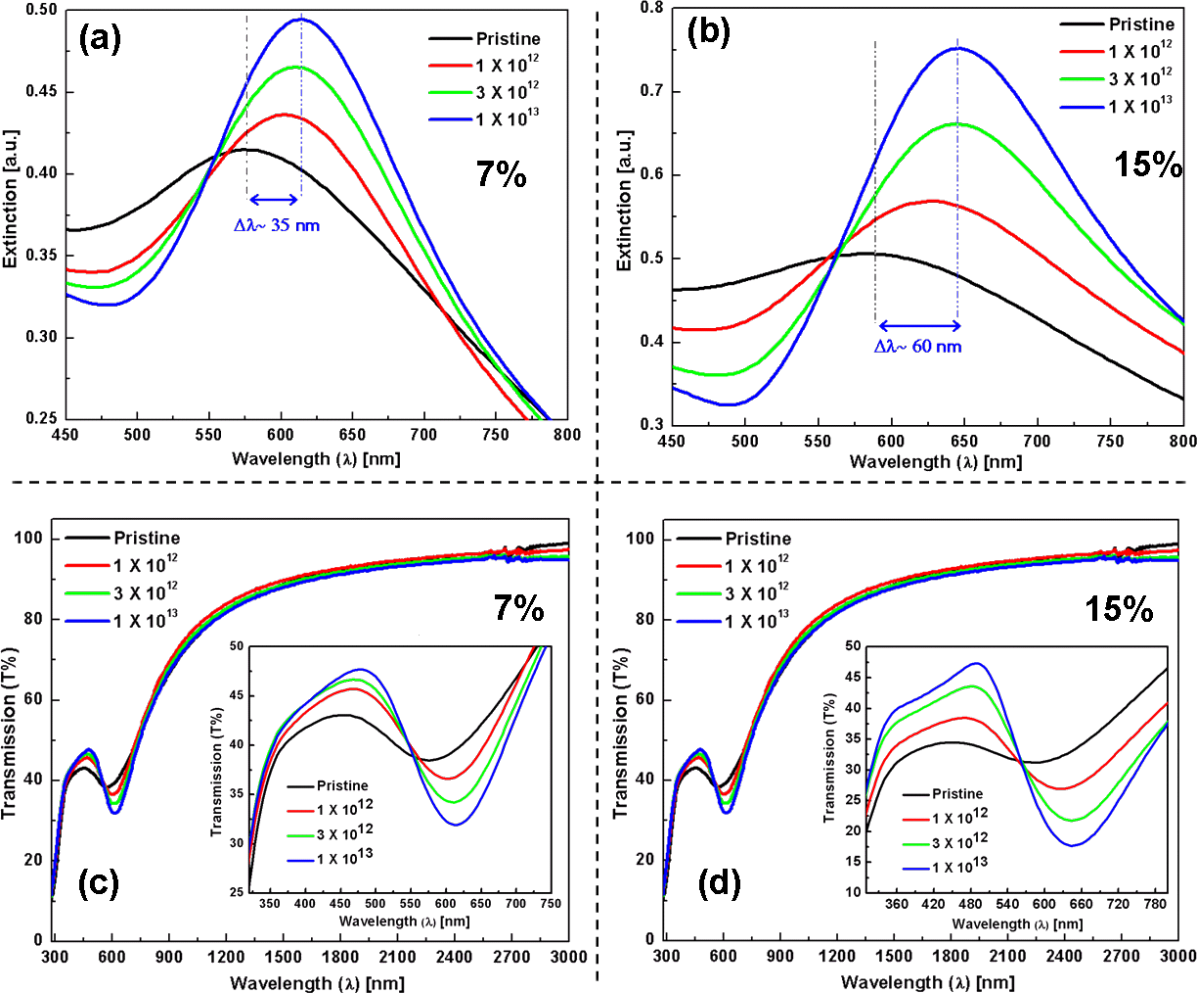
UV–visible absorption and transmission spectra of Au–TiO_2_ nanocomposite films (with an MVF of about 7% and 15%, respectively) at different ion beam fluences (1 × 10^12^, 3 × 10^12^ and 1 × 10^13^ ions/cm^2^). (a,b) Variations in SPR absorption as a function of the ion beam fluence. (c,d) Transmission spectra of Au–TiO_2_ nanocomposite films (corresponding to a and b) as a function of the ion beam fluence. The inset images (in c and d) show the magnified views from band-edge regions.

The optical properties of pristine as well as irradiated Au–TiO_2_ nanocomposite films (with an MVF of about 7% and 15%) have been measured by using UV–visible spectroscopy and are discussed here. Figure 6 shows the SPR absorption spectra (a,b) and transmission spectra (c,d) of nanocomposite films with MVF ≈ 7% and 15%, respectively. After irradiation (up to 1 × 10^13^ ions/cm^2^), the UV–visible spectra for both nanocomposites show a red shift of the SPR peak position. The shift of the SPR peak is larger for the nanocomposite film having a higher MVF (Δλ ≈ 35 nm for MVF ≈ 7% and Δλ ≈ 60 nm for MVF ≈ 15%, respectively). The transmission spectra for irradiated Au–TiO_2_ nanocomposite films show that the transmission behavior for both nanocomposite films is only affected in the vicinity of TiO_2_ and SPR band-edges. For higher wavelengths (beyond the tail of SPR absorption) the nanocomposites are almost transparent.

A closer look at the spectra in Figure 6a,b suggest that each spectrum mainly consists of two types of information, i.e., i) band-edge at lower wavelength (~320 nm) which is due to TiO_2_ matrix and ii) a peak in the visible–near infrared region (from about 580 nm to 650 nm for the different spectra) that arises from surface plasmon resonance absorption due to electron density oscillations in Au nanoparticles induced by electric field vector of light. From Figure 6, it can be clearly observed that SHI irradiations induce significant changes in the Au–TiO_2_ nanocomposite. With an increase in ion fluence the band-edge of TiO_2_ matrix shifts to lower wavelengths, which indicates an improvement in crystallinity of the matrix. From Tauc plot analyses for both nanocomposites, a shift of ca. 0.1 eV in the band-edge energy of TiO_2_ (between pristine and 1 × 10^13^ ions/cm^2^) is observed, which also confirms the structural changes in the TiO_2_ matrix. The red shift of the SPR peak, a slight narrowing of full width at half maximum (FWHM) and a simultaneous increase in SPR peak intensity with an increase in ion fluence are clear indications for the growth of Au nanoparticles in the nanocomposite film.

**Figure 7:**
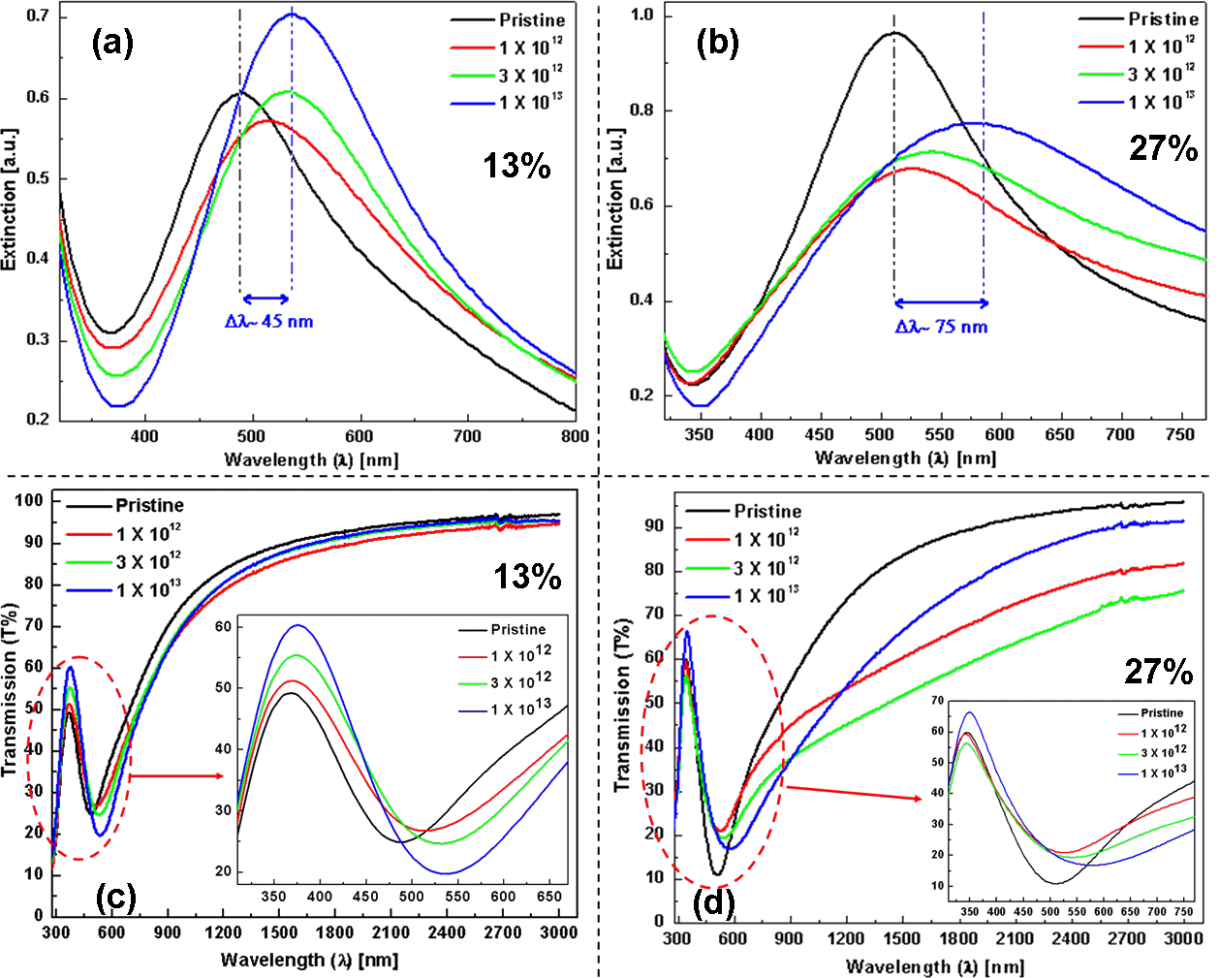
(a, b) UV–visible absorption spectra of Ag–TiO_2_ nanocomposite films (MVF ca. 13% and 27%, respectively) as a function of the ion beam fluence (from 1 × 10^12^ to 1 × 10^13^ ions/cm^2^). A red shift of the SPR peak of about 45 nm and 75 nm (for 13% and 27%, respectively) is observed after irradiation at the highest fluence. (c, d) Transmission spectra of Ag–TiO_2_ nanocomposite films as a function of the ion beam fluence. Inset images (in c and d) are the magnified views corresponding to marked regions.

Similar to Au–TiO_2_ nanocomposites, detailed UV–visible absorption and transmission studies for SHI-irradiated Ag–TiO_2_ nanocomposites (MVF ca. 13% and 27%) at different fluences (1 × 10^12^, 3 × 10^12^ and 1 × 10^13^ ions/cm^2^) were performed and the corresponding results are shown in Figure 7. The variation in plasmonic response of these Ag–TiO_2_ nanocomposites are shown in Figure 7a,b and a red shift (Δλ ≈ 45 nm for 13% and Δλ ≈ 75 nm for 27%, respectively) of the SPR peak has been observed after irradiation at 1 × 10^13^ ions/cm^2^ fluence. The optical behaviour of the Ag–TiO_2_ nanocomposite system is quite different as compared to Au–TiO_2_ in terms of structural changes in the TiO_2_ matrix (band-edge shift from Tauc plots are given in Supporting Information File 1, Figure S2 and Figure S3) and SPR peak positions after SHI irradiation. The shift of the band-edge of the TiO_2_ matrix is very small and the SPR peaks have broadened (become larger with increase in MVF) after SHI irradiation. The SPR peak intensity for the nanocomposite film with lower volume fraction remains almost unchanged up to a fluence of 3 × 10^12^ ions/cm^2^ and increases (Figure 7a) for the highest fluence. However, for the nanocomposite film with MVF ≈ 27%, a decrease in the SPR peak intensity is observed after ion irradiation. The transmission spectra of the Ag–TiO_2_ nanocomposite films are shown in Figure 7c,d and it can be observed that trend is almost similar to that of Au–TiO_2_ nanocomposites. However, the behavior in vicinity of the band-edges (TiO_2_ and SPR) is quite different. The broadening of the SPR peaks of the Ag nanoparticles also affects the transmission behaviour of these nanocomposites. The change in transmission is almost negligible for the nanocomposite film with MVF ≈ 13%. However, with increase in MVF a reduction can be observed at different ion fluences.

The plasmonic behavior of metallic nanoparticles embedded in the nanocomposite films mainly depends on the following factors: i) morphology, IPS, size distribution of nanoparticles, and ii) the dielectric constant of the host matrix (TiO_2_ in present case). It has already been demonstrated that the pristine nanocomposite films (Au–TiO_2_ and Ag–TiO_2_) in the present case exhibit bimodal particle size distributions [14] and that the TiO_2_ matrix is amorphous (evident from SAED patterns). For bimodal particle size distribution, the detailed TEM analysis has demonstrated that big nanoparticles are on top of the surface, while the smaller ones are embedded inside the nanocomposite film [39–40]. In principle, one should observe a double SPR peak corresponding to the bimodal size distribution of the nanoparticles. But in the case studied here, since the number of larger nanoparticles is very low as compared to that of smaller ones, only one broad SPR peak is observed. It is very important to mention the dependence of the SPR on these parameters because under swift heavy ion irradiation all these parameters (size of nanoparticles, size distribution, and refractive index of TiO_2_ matrix) are modified. Earlier studies have demonstrated that swift heavy ion irradiation can result in the reduction or growth of nanoparticles depending upon their size and inter-particle separations in the nanocomposite films [24]. In the Au–TiO_2_ and Ag–TiO_2_ nanocomposites studied here, the inter-particle separation is relatively small and, hence, the growth of nanoparticles has been observed after SHI irradiation as evident by TEM results and red shifts in SPR peaks. With increasing size of the nanoparticles, the SPR peak shows red shift. But, generally, the peak shift is not very large (Figure 6 and Figure 7). However, an increase in the refractive index of the matrix contributes to a large shift of the SPR peak position of the nanoparticles [47]. Earlier studies about SHI-induced modifications in TiO_2_ thin films have reported structural transformations as well an increase of the dielectric constant [35]. The increase of the dielectric constant is a direct consequence of the increase in refractive index of the host matrix and contributes significantly to the red shift of the SPR peak positions. The area under the SPR curve is measure for the total number of nanoparticles present in the nanocomposite film. As mentioned above, there are, apart from nanoparticles visible in TEM, numerous atoms, clusters and smaller nanoparticles in the as-deposited films, which contribute to the further growth of new nanoparticles as well as an increase in the size of already existing nanoparticles. Therefore, after SHI irradiation, the number of nanoparticles is most likely increased which could also be responsible for the enhanced SPR absorption peak (Figure 6a,b and Figure 7a). As long as there are atomic species available to participate in nucleation and growth, the number of nanoparticles in the nanocomposite film will continuously increase with increasing ion beam fluence. When the irradiation fluence is increased beyond a certain threshold (so that almost all metallic species are consumed after irradiation), the resultant number of nanoparticles present in the nanocomposite film might decrease due to agglomeration of smaller nanoparticles into bigger ones as higher fluences directly correspond to a larger amount of thermal energy deposited in the nanocomposite film. For nanocomposite films with higher metal volume fractions, the growth behavior of the nanoparticles under SHI irradiation might be different as observed by the reduction in SPR intensity for the Ag–TiO_2_ nanocomposite with MVF ≈ 27% in Figure 7b. When the metal volume fraction is high, there is a high probability for the formation of irregularly shaped Ag nanoparticles with decreased inter-particle distances (a tendency towards percolation). This will enhance the plasmonic coupling between the nanoparticles and lead to a broadening of the SPR peak which can be observed in Figure 7b. It is very important to mention here that for the TEM investigations the specimens were deposited on TEM grids, while for the SPR measurements glass substrates have been used. Therefore it will be difficult to correlate the total number of nanoparticles from the TEM size distribution with the observed SPR enhancements after SHI irradiation at different fluences. Therefore, the observed red-shifts of the SPR positions in the Au–TiO_2_ and Ag–TiO_2_ nanocomposite films studied here are due to cumulative effects from an increase in particle size, a change in the size distribution and, most significantly, because of structural changes in the host TiO_2_ matrix.

**Figure 8:**
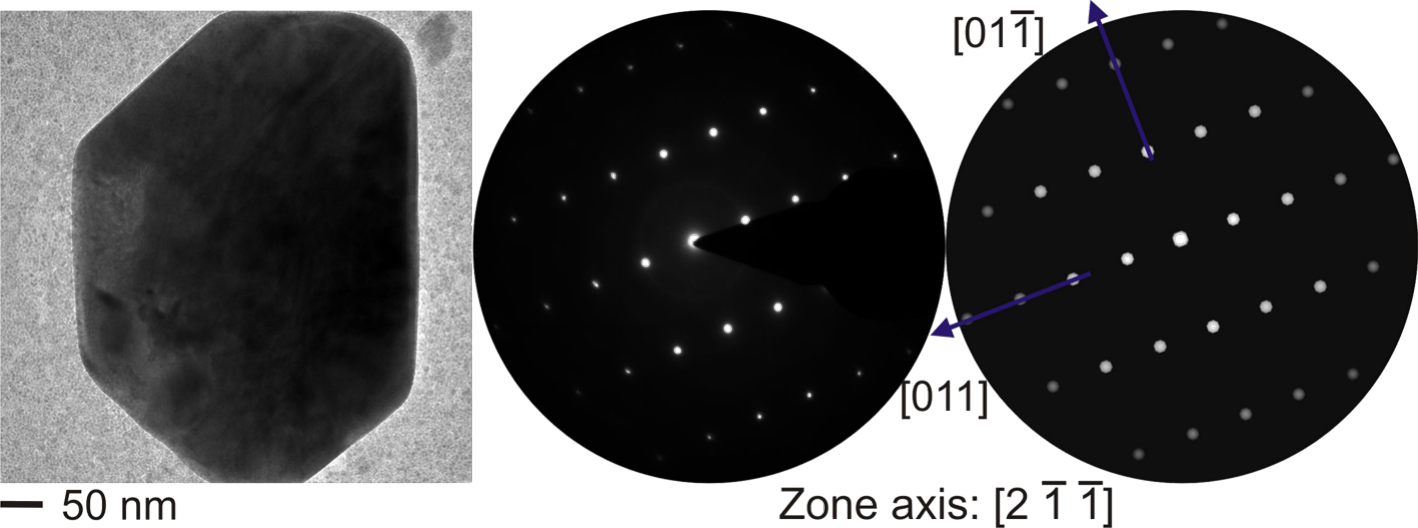
Micron-sized single crystalline TiO with the corresponding experimental SAED pattern and simulated SAED pattern.

During TEM measurements of the Ag–TiO_2_ nanocomposite (MVF ≈ 13%) irradiated at 3 × 10^12^ ions/cm^2^, the formation of some large sub-micron sized crystals with various morphologies were observed [48]. In addition, small TiO*_x_* fragments were found at higher fluences. Since growth of Ag nanoparticle with such a large dimension was unexpected, detailed TEM investigations on these large particles were performed and measurements revealed that they were TiOx crystals. The TEM, SAED, and corresponding simulation pattern is shown in Figure 8. The detailed TEM, SAED, and EDAX studies confirmed the formation of crystalline TiO phase after SHI irradiation.

The formed crystals are of the order of 400 nm in size and exhibit a similar *d*-spacing as reported for TiO by Bartkowski et al. [49]. However, there are only very few reports, which describe the formation of TiO nanostructures through various methods [43–44]. Hence the fact that, in the present case, the formation of this phase briefly occurs and then vanishes again with increasing fluence can only be understood by the interaction of two different counteracting mechanisms evolving at different fluences. According to this postulation, at lower fluences, one observes the tendency towards the formation of TiO, with larger unaffected area. At higher fluences, one can see the destruction of the evolved TiO phase into fragments. According to this supposition, the emergence of double or multiple hits signifies phase destruction, the further increase in fluence leads to the destruction of that previously created TiO phase. From the SAED patterns (Figure 8), the [2 −1 −1] zone axis of the TiO phase agrees with the simulated results for TiO by using the JEMS software [50].

**Figure 9:**
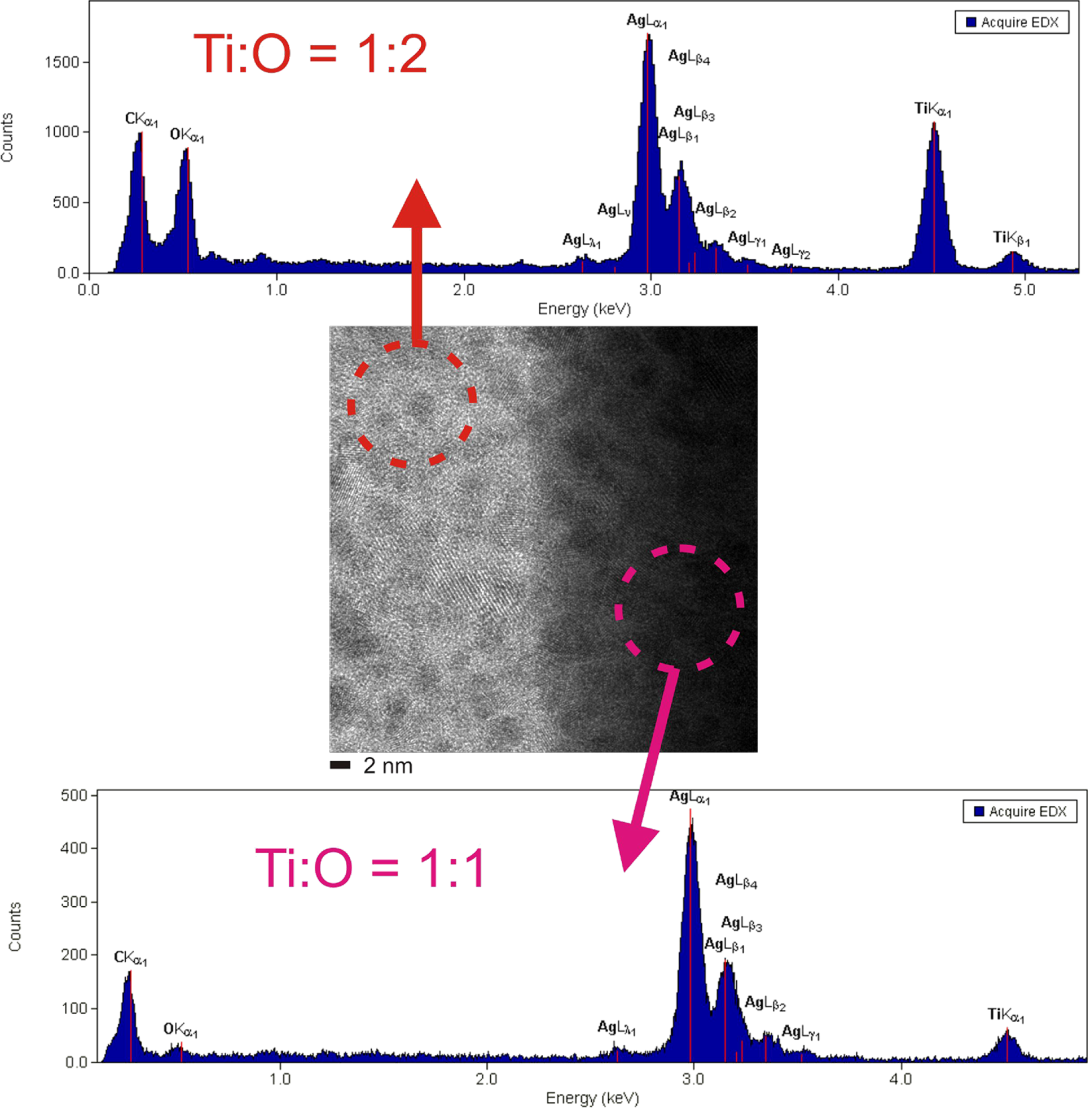
TEM nanoprobe EDX analysis on the TiO crystal and the matrix confirming the ratio of Ti:O in both the areas to be 1:1 and 1:2, respectively.

In spite of the well-known problems of the light elements' quantification by EDX, test measurements on distinct samples (e.g., amorphous TiO_2_) point to a sufficient reliability of the setup for a semi-quantitative interpretation. Hence, the EDX-nanoprobe analysis of the TEM (Figure 9) confirms that the ratio of Ti:O in the nanocomposite is 1:2 and that it is 1:1 in the nanocrystal. Interestingly, the desired equimolar ratio of Ti and O is well adjusted even on the nanoscale. But in order to confirm this, further SHI irradiation studies on these nanocomposites are required to be performed in a systematic manner and the same will be planned in future.

**Figure 10:**
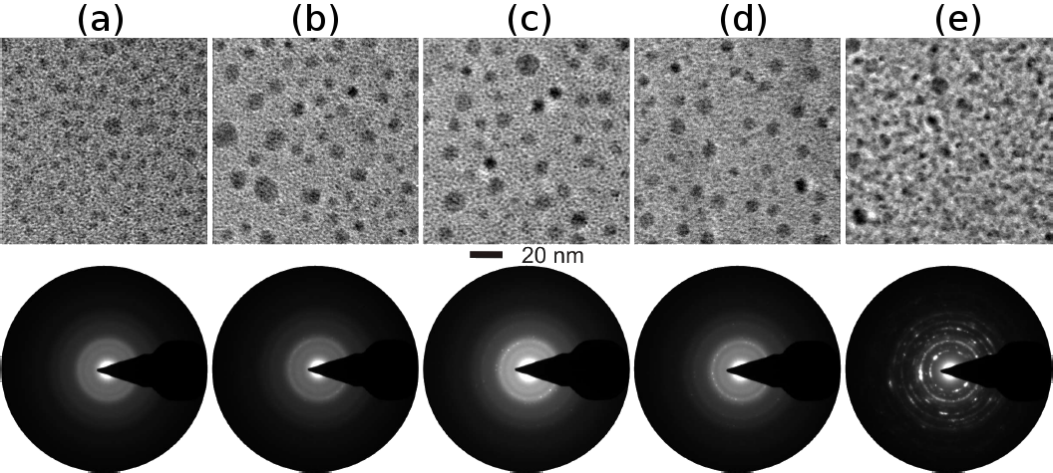
In situ heating of the Ag–TiO_2_ nanocomposites at (a) room temperature (b) 150 °C, (c) 300 °C, (d) 400 °C and (e) 500 °C (total time: 3 h).

The formation of TiO nanostructures in the Ag–TiO_2_ nanocomposites is only possible by SHI irradiation (this process is far from thermodynamic equilibrium) as compared to conventional heating experiments (in thermodynamic equilibrium) and it was also revealed by a comparative study involving the in situ heating of the Ag–TiO_2_ nanocomposites in the TEM. From the in situ TEM heating experiments (Figure 10), crystallization of the matrix with the associated growth of the nanoparticles was observed.

On in situ heating, from room temperature to 500 °C, there is an increase in the size of the nanoparticles due to Ostwald ripening, (also observed after the in situ heating of Au–TiO_2_ nanocomposites). In addition, evidence for the changes in the matrix at 500 °C can also be observed in the SAED patterns. Although signatures for the change in the matrix are evident right from 300 °C through the diffuse intensities corresponding to the reflections of the anatase form of TiO_2_. At 500 °C, these appear as ring patterns confirming the crystallization of TiO_2_ into the anatase type.

## Conclusion

In conclusion, we have successfully fabricated Ag–TiO_2_ and Au–TiO_2_ nanocomposites with desired metal volume fractions in a controlled manner. The microstructural evolutions in the nanocomposite films by detailed TEM analysis revealed the bimodal size distribution of metal nanoparticles in as-deposited nanocomposite films with larger nanoparticles on the surface and smaller nanoparticles embedded inside the nanocomposite film. SHI irradiation of these nanocomposite films at different fluences resulted in an improvement in the crystalline nature of host TiO_2_ matrix as well as growth in the average diameter of nanoparticles. Formation of different phases of the host TiO_2_ matrix is also observed under SHI irradiation which is most likely due to structural transformations due to large amount of electronic energy deposited into the nanocomposite films. The growth of nanoparticles in the metal–titania nanocomposite films under swift heavy ion irradiation has been discussed in terms of dissolution and growth induced by large electronic energy deposition. The deposited thermal energy is sufficient to promote the growth of nanoparticles and the structural changes in the TiO_2_ matrix. With increase in ion beam fluence, the growth of larger nanoparticles has been observed. Plasmonic properties of Au–TiO_2_ and Ag–TiO_2_ nanocomposite films before and after SHI irradiations always showed a red shift of the SPR peak position after irradiation. The red shift of the SPR peaks in the both nanocomposite films has been explained in terms of growth in size of nanoparticles as well structural transformations in the host TiO_2_ matrix.

## Experimental

Ag–TiO_2_ and Au–TiO_2_ nanocomposite thin films were prepared by co-sputtering from two different magnetron sources in a home-made vacuum deposition chamber. The host matrix (TiO_2_) and metal (Ag/Au) targets were co-sputtered by using two different magnetron sources, i.e., RF and DC, respectively, in the chamber. The deposition chamber was evacuated to a base pressure of 10^−7^ mbar with the help of a rotary pump (for pre-vacuum) followed by turbo molecular pump (for high vacuum). Metal was deposited by the DC planar magnetron source ION’X 2UHV (Thin Film Consulting). A similar-type RF magnetron source was used for sputtering the copper-bonded titanium dioxide (Williams Advanced Materials) to prevent charging of the target. The deposition rates from both targets were in situ monitored by two independent quartz-crystal monitors. For TiO_2_, the deposition rate was varied from 1 to 4 nm/min by varying the RF power, while in the case of Au/Ag, the deposition rates were varied from 0.5 to 3 nm/min by changing the DC power. The metal volume fractions of the nanocomposite films were monitored by controlling the deposition rates of metal and matrix, respectively. The sample holder was rotated throughout the deposition process to achieve uniform and homogeneous deposition of all the samples mounted on the sample holder. The thickness of the deposited films was measured by a surface proﬁlometer (Dektak 8000) by depositing the nanocomposite film on a masked silicon wafer. Subsequently, the metal volume fractions in the nanocomposite films were also determined by using energy dispersive X-ray spectrometer (SEM–EDX Philips XL30) with proper calibration. For characterization convenience, these nanocomposite films were simultaneously deposited at different substrates, e.g., glass (for UV–visible absorption), carbon-coated Cu grids (for TEM measurements) and Si substrates for EDX. The deposited Ag–TiO_2_ and Au–TiO_2_ nanocomposite films with different MVFs (for gold: 7% and 15%, for silver: 13% and 27%) were irradiated by 100 MeV Ag^8+^ ions at different fluences (1 × 10^12^, 3 × 10^12^, 1 × 10^13^ ions/cm^2^) by using the Pelletron accelerator facility at Inter University Accelerator Centre, New Delhi. The energy of the Ag ions was selected by “Stopping and range of ions in matter (SRIM) 2008” calculations [51]. The values of electronic energy loss (*S*_e_) for 100 MeV Ag^8+^ ions in Au–TiO_2_ (MVF ≈ 15%) and Ag–TiO_2_ (MVF ≈ 13%) nanocomposites are about 14.9 and 13.9 keV/nm, respectively (Supporting Information File 1, Figure S1). The values of the corresponding nuclear energy losses for both of the cases are very small and can be neglected. Since the nanocomposite film thicknesses are very small, the *S*_e_ value can be assumed to be uniform all along the film thickness. To investigate the effect of ion irradiation, detailed characterizations of pristine as well as irradiated nanocomposite films on different substrates have been performed. The microstructural evolution of nanoparticles as well as of the host matrix in the nanocomposite films have been investigated by transmission electron microscopy (Philips Tecnai F30 G^2^). Optical extinction studies of the nanocomposite films were carried out by using a UV–vis–NIR spectrophotometer (Perkin Elmer Lambda 900).

## Supporting Information

File 1 Additional experimental data
