# Supplementary material for: Microstructural and plasmonic modifications in Ag–TiO2 and Au–TiO2 nanocomposites through ion beam irradiation
Source: Beilstein J Nanotechnol. 2014 Sep 1;5:1419–31. doi: 10.3762/bjnano.5.154 (PMC4168693; doi:10.3762/bjnano.5.154)
Supplement: File 1 — Additional experimental data [file Beilstein_J_Nanotechnol-05-1419-s001.pdf]

## Supporting Information

for

# Microstructural and plasmonic modifications in Ag–$TiO_2$ and Au–$TiO_2$ nanocomposites through ion beam irradiation

Venkata Sai Kiran Chakravadhanula[§1], Yogendra Kumar Mishra[2], Venkata Girish Kotnur[¶1], Devesh Kumar Avasthi[3], Thomas Strunskus[1], Vladimir Zaporotchenko[1], Dietmar Fink[4], Lorenz Kienle[5] and Franz Faupel[1*]

Address: [1]Chair for Multicomponent Materials, Institute for Materials Science, Christian Albrechts University Kiel, Kaiserstr. 2, Kiel, 24143, Germany; [2]Functional Nanomaterials, Institute for Materials Science, Christian Albrechts University Kiel, Kaiserstr. 2, Kiel, 24143, Germany; [3]Inter University Accelerator Center, Materials Science Group, P.O. Box: 10502, New Delhi, 110067, India; [4]Instituto da Fisica, Universidad Autonoma Metropolitana–Iztapalapa, Av. San Rafael Atlixco No. 186, Col. Vicentina, Delegacion Iztapalapa, Mexico D.F., 09340, Mexico and [5]Synthesis and Real Structure, Institute for Materials Science, Christian Albrechts University Kiel, Kaiserstr. 2, Kiel, 24143, Germany.

[§] Presently at Helmholtz Institute Ulm (HIU) Electrochemical Energy Storage, Albert-Einstein-Allee 11, 89081 Ulm, Germany.

[¶] Presently at Precision and Microsystems Engineering, Delft University of Technology, Mekelweg 2, 2628 CD DELFT, The Netherlands.

Email: Franz Faupel* - ff@tf.uni-kiel.de

* Corresponding author

## Additional experimental data

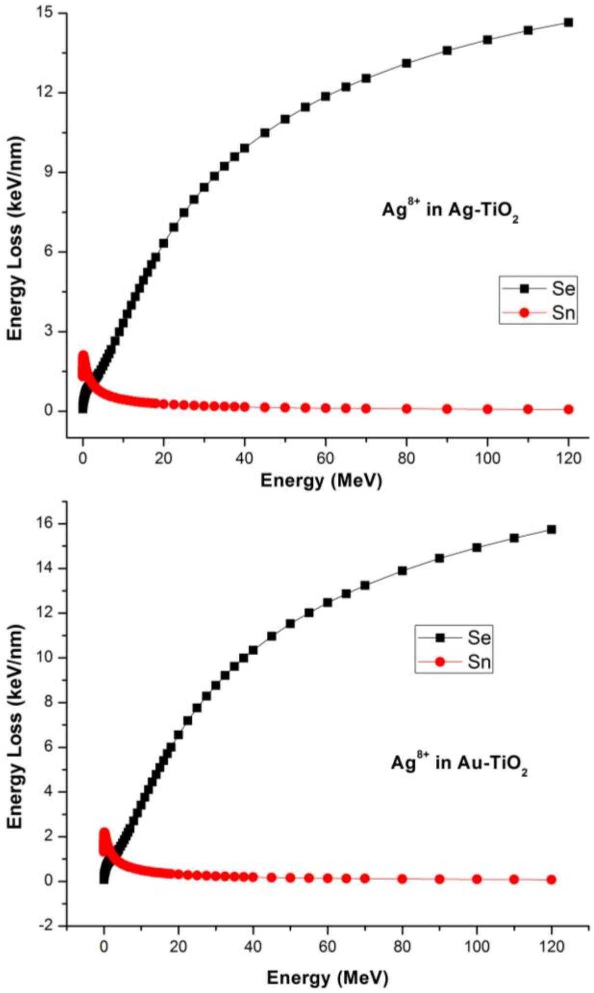


**Figure S1:** Simulated electron energy loss of $Ag^{8+}$ ions in Ag–$TiO_2$ and Au–$TiO_2$ nanocomposites [1].

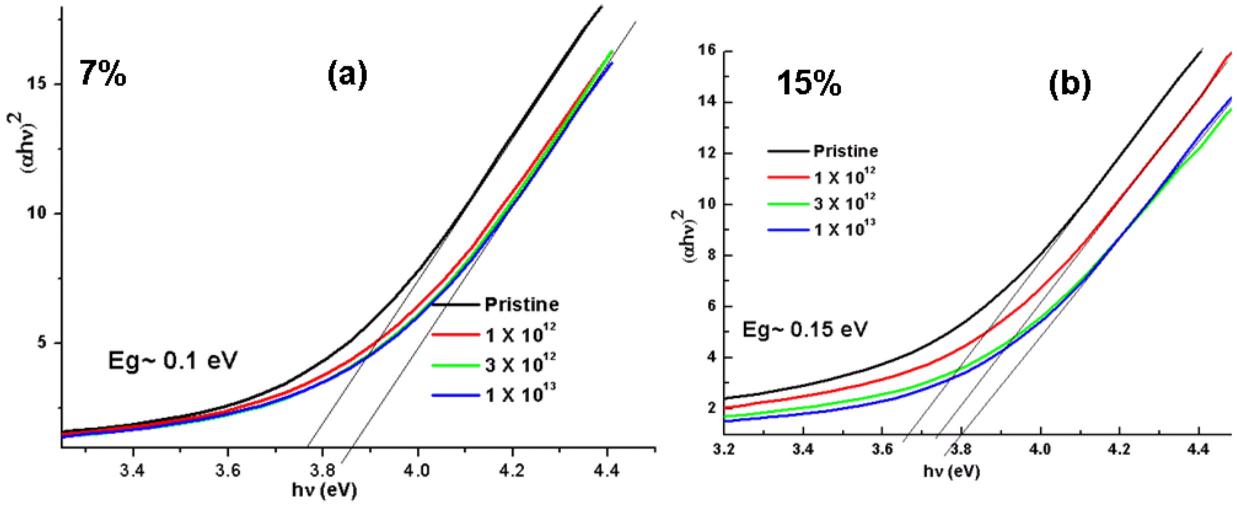


**Figure S2:** Bandgap analysis (Tauc plot) of pristine and irradiated (up to $1 \times 10^{13}$ ions/cm$^2$) Au–$TiO_2$ nanocomposites, (a) metal volume fraction ca. 7%, (b) metal volume fraction ca. 15%. In case of 7 %, an increase in bandgap of about 0.1 eV (a) however for 15 % nanocomposite film, this shift is around 0.15 eV.

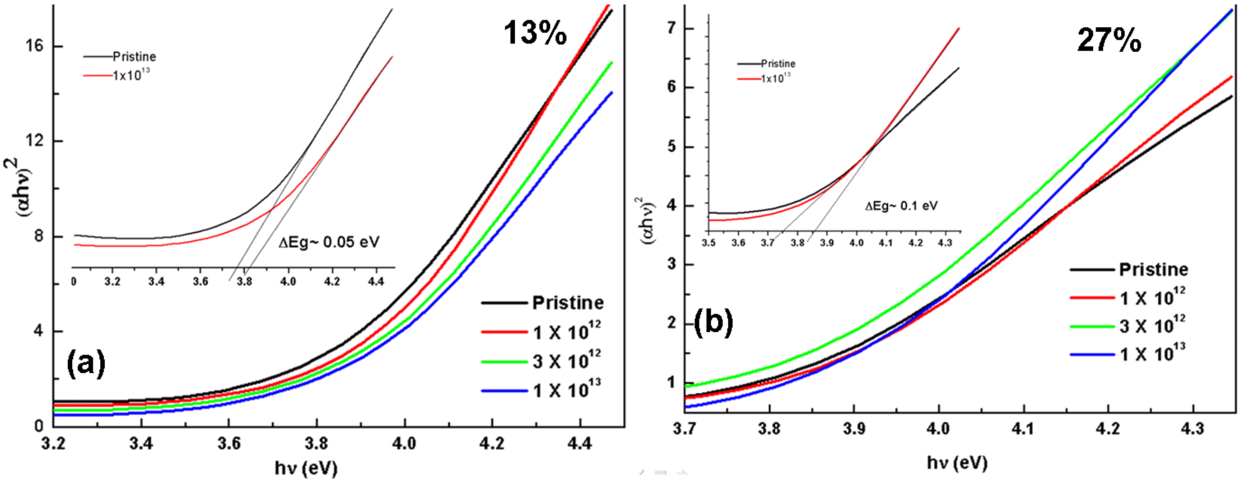


**Figure S3:** Bandgap analysis (Tauc plot) of pristine and irradiated (up to $1 \times 10^{13}$ ions/cm$^2$) Ag–$TiO_2$ nanocomposites, (a) metal volume fraction ca. 13%, (b) metal volume fraction ca. 27%. In both cases, the band-edge shift is irregular which is also evident for SPR variation.

**References**

[1] James Ziegler - SRIM & TRIM. http://www.srim.org/ (accessed May 11, 2014).
